# Using cementochronology to assess the seasonality of catastrophic events in medieval mass graves (Kutná Hora-Sedlec, Czechia, 14^th^ century): Preliminary results

**DOI:** 10.1371/journal.pone.0295757

**Published:** 2023-12-13

**Authors:** Eliška Zazvonilová, Hana Brzobohatá, Jan Frolík, Petr Velemínský, Jaroslav Brůžek

**Affiliations:** 1 Institute of Archaeology of the Czech Academy of Sciences, Prague 1, Czech Republic; 2 Faculty of Science, Department of Anthropology and Human Genetics, Charles University, Prague 2, Czech Republic; 3 Department of Anthropology, National Museum, Prague 1, Czech Republic; UNCG: University of North Carolina at Greensboro, UNITED STATES

## Abstract

While season-of-death estimation using cementochronology is routine in archaeozoology, its use is much less frequent in bioarchaeology. Based on the character of the outermost increment (bright or dark), two seasons (spring/summer, autumn/winter) can be distinguished. Although many studies mention its potential and possible use in forensic anthropology or bioarchaeology, few exist with estimation results. This study aimed to apply cementochronology–a histological method based on counting and assessing regular circa-annual acellular cementum increments–to 42 individuals from medieval mass graves from Kutná Hora-Sedlec (Czechia, 14^th^ century) to estimate the season-of-death. The mass graves belong to two stratigraphically distinct groups; written and archaeological sources relate them to two catastrophic events (the famine of 1318 and the plague epidemic of 1348–1350). Using cementochronology, we distinguished two distinct seasons corresponding to the two groups of graves, with individuals from the first group dying predominantly in spring/summer, while those from the second group died in autumn/winter. Taking into account the typical seasonal dynamics of epidemics, the results would be more in line with written sources. However, during the evaluation, we faced difficulties identifying the outermost increment and detecting the dark (thinner) increment; we recommend including only young and middle-aged adults in future studies, due to the difficulty of evaluation, and to consider the readability of the tissue (often affected by diagenesis). In conclusion, cementochronology has potential in the context of estimating the season-of-death, but the technical possibilities for enhancing the outermost increment need to be addressed, and the amount of data analysed expanded.

## Introduction

### Reconstructing seasonality in bioarchaeology

Seasonality in a human-environmental relationship is an important and complex issue in bioarchaeology [1]. The reconstruction of seasonal patterns finds application in topics such as the seasonality of particular activities, occupation of sites, or function of some particular sites [2–5]. Methods for investigating past seasonality, such as isotope analyses or the study of the microstructure of tooth cementum, can also provide a deeper understanding particularly of shorter-term activities in comparison to radiocarbon dating [1]. The study of the microstructure of tooth cementum using the method of cementochronology has a long tradition, and not only in archaeology [6]. This histological method is based on the seductive principle of the circa-annual cementum increments in the acellular tooth cementum [7]. Under light microscopy, each pair of light and dark bands represents one year [8,9]. The season-of-death can be assessed based on the outermost increment –a bright thicker increment represents spring/summer, and a dark thinner band the autumn/winter months [8,10]. Compared to archaeozoology, where cementochronology is routinely used for age and season-of-death estimation, in anthropology, the method is not often used, and if so, for age estimation, with a few studies on the season-of-death assessment [e. g., 11–13]. Preserved teeth allow season-of-death estimation not only in cases of the topics mentioned above, but also in another important topic related to seasonality–episodes of famine or infectious diseases.

### Catastrophic events in medieval Kutná Hora-Sedlec

Catastrophic events such as famine or infectious diseases were not exceptional in Europe during the Middle Ages. Kutná Hora-Sedlec (Czechia) represents the most extensive set of medieval mass graves relating to catastrophic events on a European scale [14]. During the reconstruction of a church with an ossuary, 32 complete and incomplete mass burials (with around nine hundred skeletons) and 878 individual graves were excavated [14,15]. Kutná Hora-Sedlec has been intensively studied from the archaeological [15,16] and the bioarchaeological and palaeodemographic [14,17,18] perspectives. The chronology of the mass graves at the ossuary is based on an investigation of the stratigraphic configuration, with two levels of pits [15]. Younger mass graves have been preliminarily assigned to the fatalities of the 1348–1350 plague epidemic (based on coin recoveries from two of them), while the older level is provisionally linked to famine and the burial of victims before the gates of Sedlec in Kutná Hora, based on the 1318 report of the Zbraslav Chronicle (p. 248) [19]. The plague epidemic in the Czech lands is also mentioned in several other historical sources [20], but this area was less affected area than, for example, France or Italy [21]. This leads to fewer written records of the Bohemian plague outbreak. While written materials are invaluable sources of information that cannot be obtained from skeletal remains, the link to the plague is a preliminary result, and archaeogenetic analysis can provide a definitive confirmation. However, it can be argued that the catastrophic events which occurred at Kutná Hora-Sedlec in the 14^th^ century caused increased mortality, manifested by a change in the funerary ritual (mass graves).

### The seasonal dynamics of famine and plague

Famine and plague periods, mentioned in the historical documents, can be considered as probable causes of the mass dying at Kutná Hora-Sedlec. Estimating seasonality alone cannot solve the problem of the apparent causes of the mass graves, but it can help to contribute to understanding the differences between their two stratigraphically distinct levels. We can also get closer to interpreting the cause of mass graves by linking the results of seasonality estimation to the fact that several diseases have specific seasonal dynamics. Plague, in particular, is greatly influenced by seasonal factors [22,23]. Famine is usually defined as a discrete mass starvation event triggered by a food shortage [24,25]. The causes can be both exogenous and endogenous (e. g. extreme weather, bovine diseases, or factors internal to society, such as regulations and institutions) [26]. The complex scenario of the outbreak of famine can be demonstrated in the so-called Great Famine that afflicted Northern and Western/Central Europe between 1315 and 1322, affecting the Czech lands as well. This was recorded by chroniclers of the time, and narrative sources of Czech provenance also exist (e. g. the Chronicon Regiae Aulae, written at the Cistercian monastery of Zbraslav near Prague by Peter of Zittau (1275–1339) [19]. The Great Famine was caused by an agrarian crisis that happened in a period of climate transition: between the Medieval Climatic Optimum (lasting roughly until 1200) and the onset of a cooler period, the Little Ice Age, beginning in the early 14^th^ century, when average temperatures declined [27]. As for the season of the peak of famine mortality, we rely mainly on demographic studies of post-medieval populations and the death registers of parishes [28–30]. These sources show that demographic crises caused by widespread and severe food scarcity culminate in the Northern Hemisphere in the winter or early spring months. Plague is the only medieval epidemic for which the temporal and geographical propagation characteristics have been studied and described [23,31]. The temperature plays a significant role in the timing and growth of epidemics [32].

The data composed during the time of the Medieval Black Death suggest that mortality peaked mostly during the warmer weather months between April and October [33]. This peak mortality coincides with a seasonal peak in travel and trade during this period [23] investigated seasonal patterns and the epidemic peak timing of historical plague outbreaks (mainly from the 16-19^th^ centuries) together with its association with latitude as a rough proxy measure for climatic conditions. They found that the typical plague season differed by latitude, and that the more northerly the location, the later the epidemic mortality peak occurred. Based on the findings of [23] and the city’s latitude (49°56′54″N), Kutná Hora would fall into the category of having August–September plague mortality peaks.

Our study aimed to apply cementochronology for the season-of-death estimation in 42 individuals from five mass graves coming from two stratigraphically different levels. Two distinct seasons were expected in the examined burials, hypothetically corresponding to two catastrophic events recorded in historical documents: plague and famine periods, with different seasonal peaks in mortality. We also sought to consider technical aspects of using cementochronology in bioarchaeology, including avoidable and unavoidable issues.

## Material and methods

### Material

Thirty-two complete or incomplete mass graves have been excavated at Kutná Hora-Sedlec. For our purposes, individuals from both levels of mass graves were chosen. Since the detection of the outermost line in older individuals could be problematic (due to the high number of cementum increments), only teeth from juveniles, young and middle-aged adults were included. In terms of tooth type selection, canines were preferentially chosen because of their lower susceptibility to caries [34]. Where canines were not present, first or second incisors were used. Only healthy teeth without visible caries were included. The ages of all individuals were previously estimated based on the degree of dental attrition, and the metamorphosis of sternal rib ends and articular facets of the ilium [35–39]. Sampling took place at the place where the skeletal remains are deposited, in the Department of Anthropology of the National Museum in Prague (grave numbers are listed in the Material section and also in S1 Table). No permits were required for the described study, which complied with all relevant regulations.

**Fig 1:**
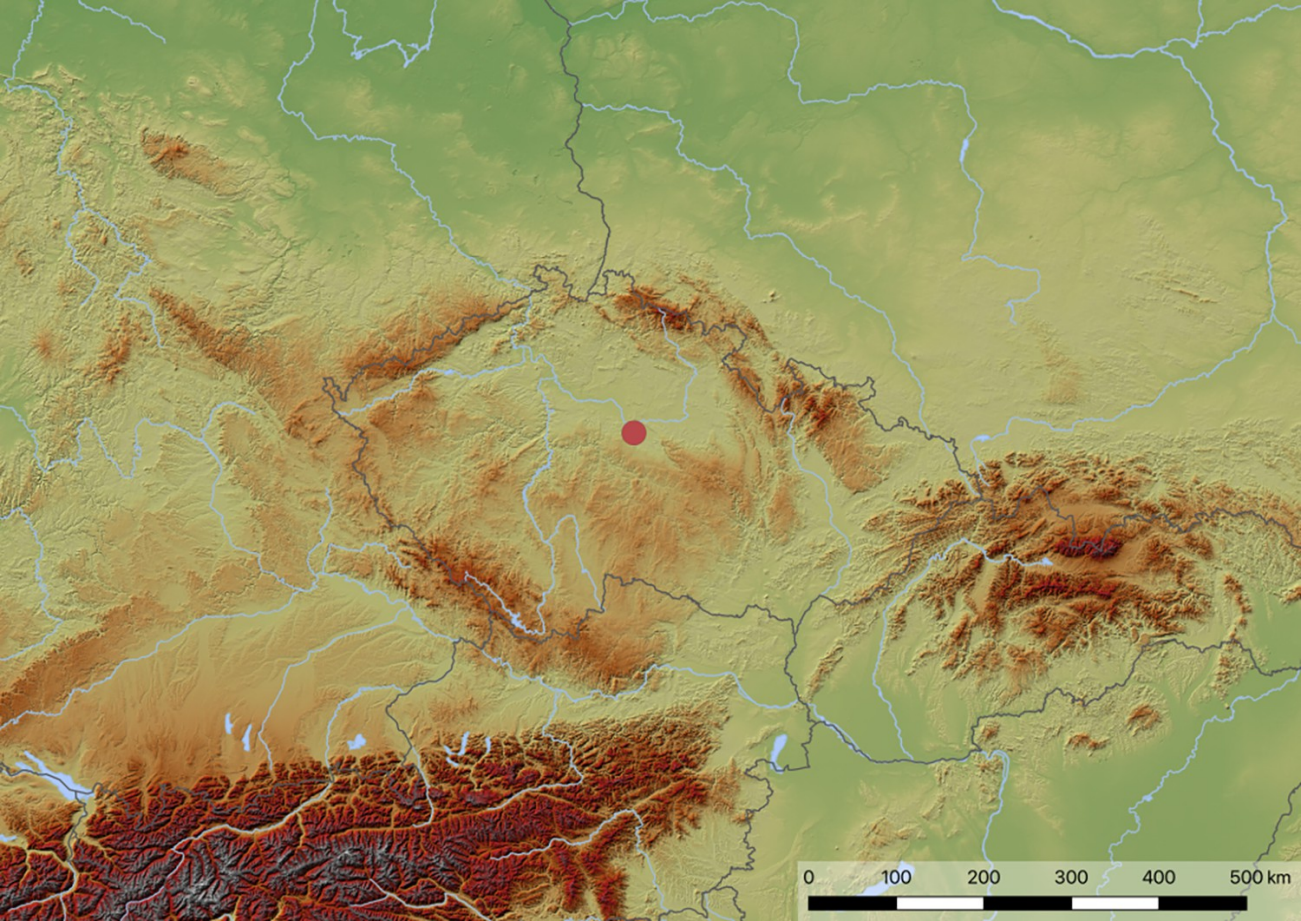
The location of Kutná Hora-Sedlec (indicated by a red dot), based on Open Maps For Europe (https://www.mapsforeurope.org), © EuroGeographics [2023].

Of the total number of 42 examined individuals, 22 belonged to two "plague" mass graves, and 20 individuals belonged to "famine" mass graves. The 5 mass graves were marked with the letters A-E (plague mass graves nos. 516 and 764 = A and B, famine mass graves nos. 765, 853 and 1578 = C, D, E) (Fig 1). Only one tooth was selected from each individual. The distribution of selected individuals relative to the total number of individuals within the mass grave is as follows: A 8/43, B 14/54, C 16/33, D 2/17, E 2/23.

## Methods

For the preparation of thin sections, the standardized protocol [40] was used. According to protocol, teeth were at first cleaned with distilled water. Epoxy resin (Araldite 2020®) was used for embedding the teeth (without the crown). Subsequently, the tooth embedded into the resin was fixed in a holder to a precision saw with a diamond blade (Isomet Buehler 1000®), and five sequential cuts (from the middle part of the root) with a thickness of 100 μm were prepared at low speeds. Sections were mounted on slides and covered with coverslips (using Canada balsam). Each section was observed using a microscope (Olympus Bx-51), using polarized light (at 200x and 400x magnification). Segments with readable cementum were captured and saved as TIFF images. These were then edited and processed in Fiji (ImageJ) software. Prior to the age or season-of-death, the quality of the tissue affecting the results should be considered [9,41,42]. Samples were therefore included or excluded based on the readability index (measuring the distinctness of the tissue, scale 0–4; [7]). Samples (thin sections) with zero values in all five sections/tooth were excluded from the sample. Diagenetic apatite crystals can create false increments which should be distinguished from true cementum [5]. This can be done by using lambda plate, which is a frequently used approach (e. g., [4,5,43]).

**Fig 2:**
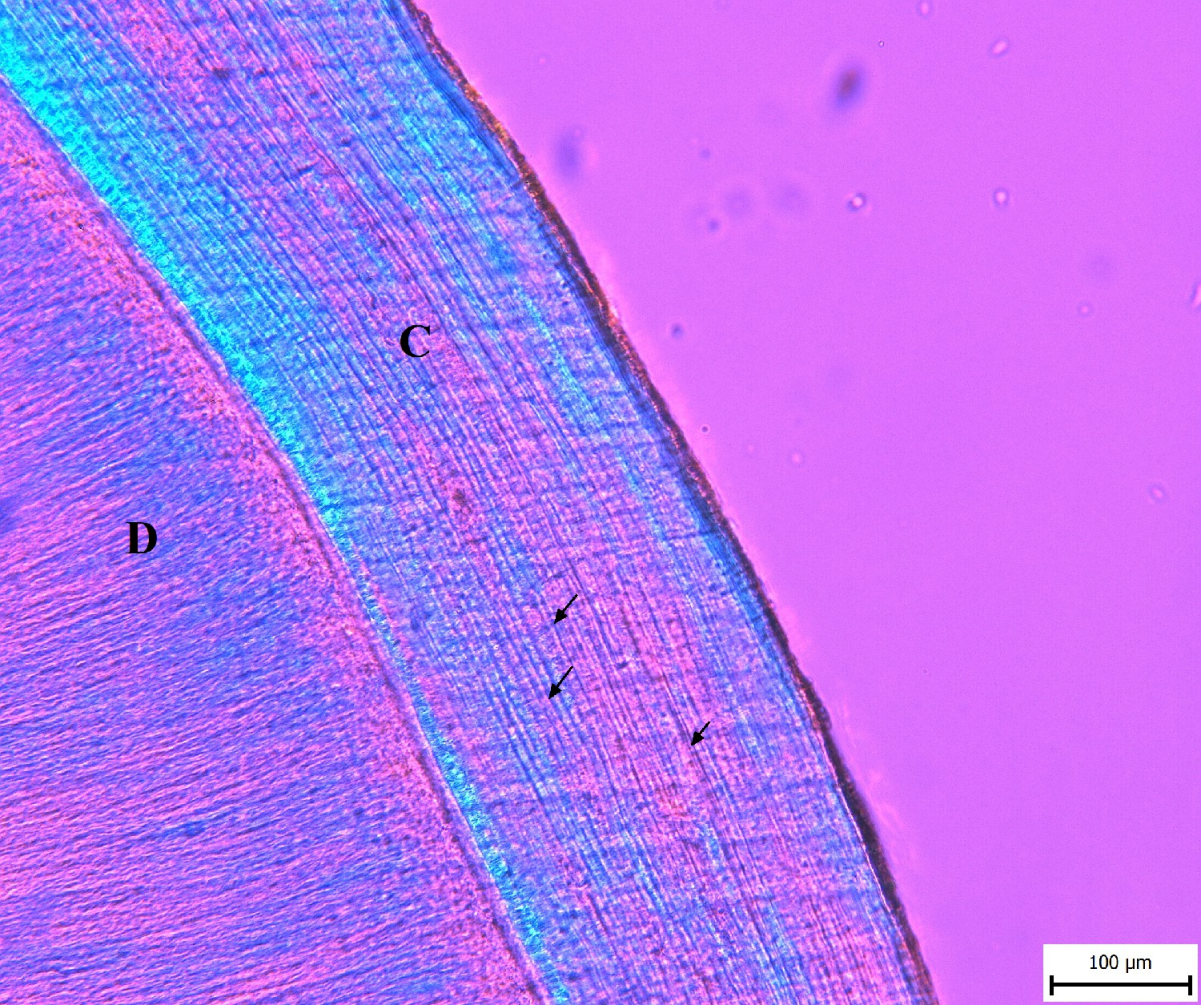
Cross-section of a tooth illustrating cementum increments (C = cementum, D = dentine) under polarized transmitted light with lambda plate. Arrows indicate opaque (dark) cementum increments.

Recording the season-of-death was performed visually, based on the saved images. After detecting the readable area of acellular cementum and distinguishing between acellular cementum and surrounding tissues, the character of the outermost increment was recorded (translucent or opaque). We assume that one increment (annual layer) consists of one pair of opaque (dark) and translucent (bright) lines [44] (Fig 2), and that the opaque (dark) line forms from late autumn to early spring, while the translucent (bright) line forms from spring to autumn [12,13,44]. When conflicting or unambiguous results were recorded, an “indeterminable” character was assigned. We then subjected our results to measures of intra- and inter-observer error, in both cases selecting ten random images that were evaluated by two observers and one observer several months apart. The results are expressed both by the verbal description in the table and by Cohen’s kappa (range of values: 0–1; 0 indicates no agreement, 1 perfect agreement).

All data were analysed in Microsoft Excel, Fiji (ImageJ) and Zoner Photo Studio X.

## Results

### Sample inclusion & exclusion

**Fig 3:**
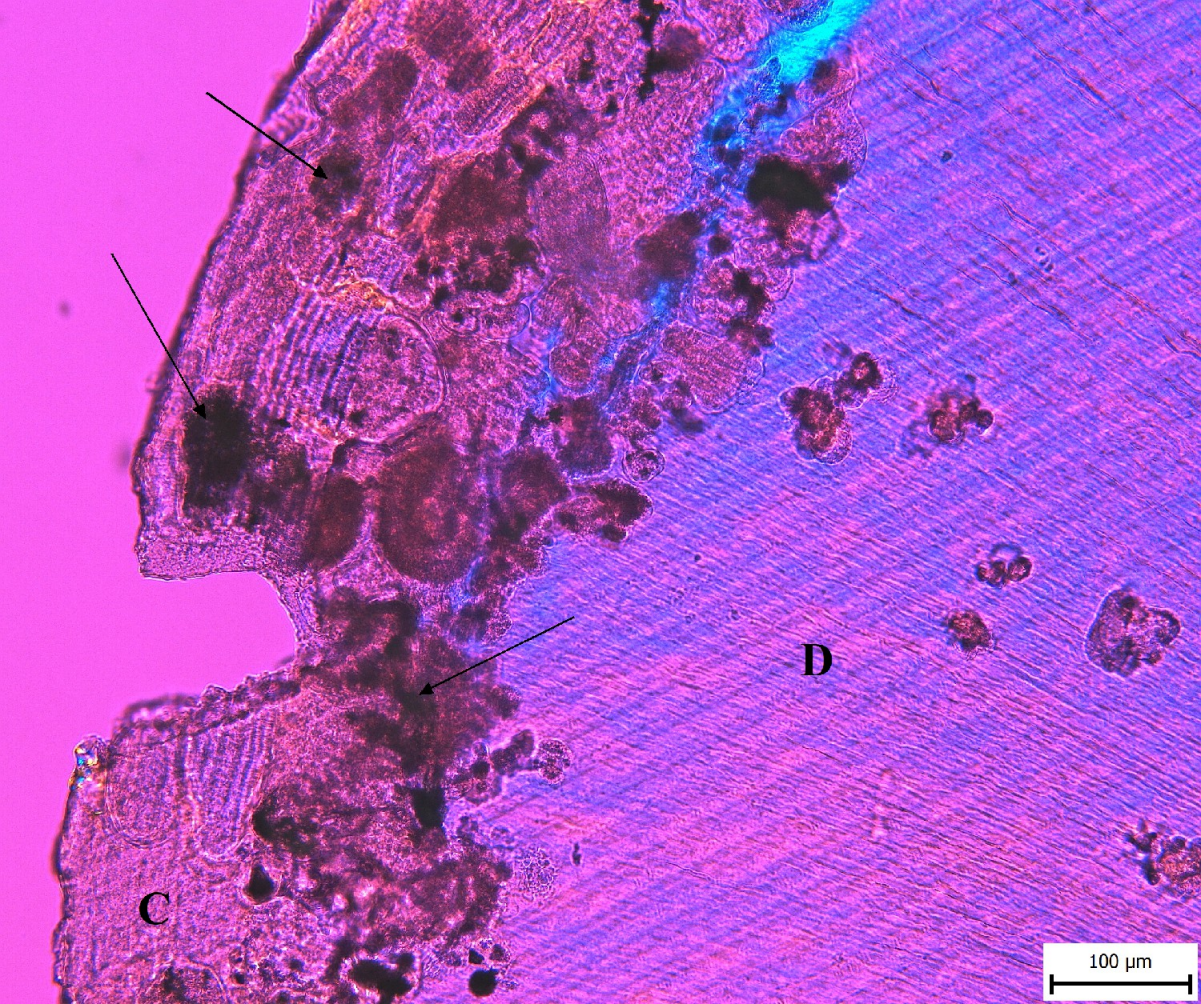
Prominent diagenetic changes affecting the readability of cementum increments. Arrows indicate destroyed areas of the acellular cementum (D = dentine, C = cementum).

Good readability of the tissue is essential for evaluating incremental lines for age or season-of-death estimation. Of the 42 teeth, 14 of them (33%) were completely excluded from sample, having zero values in all five thin sections. Mostly, prominent diagenetic changes destroyed the tissue and observing the outermost increment (or other increments) was impossible (Fig 3). In the initial phase, 14 teeth were excluded, and season-of-death estimations could be made for the remaining 28. Only in 4 teeth did all five thin sections have readable cementum enabling the estimation; of the remainder, 3/29 had four readable sections, 8/29 three readable sections, 6/29 two readable sections and 7/29 only one readable section (all results are listed in S1 and S2 Tables).

### Season-of-death estimation

**Fig 4:**
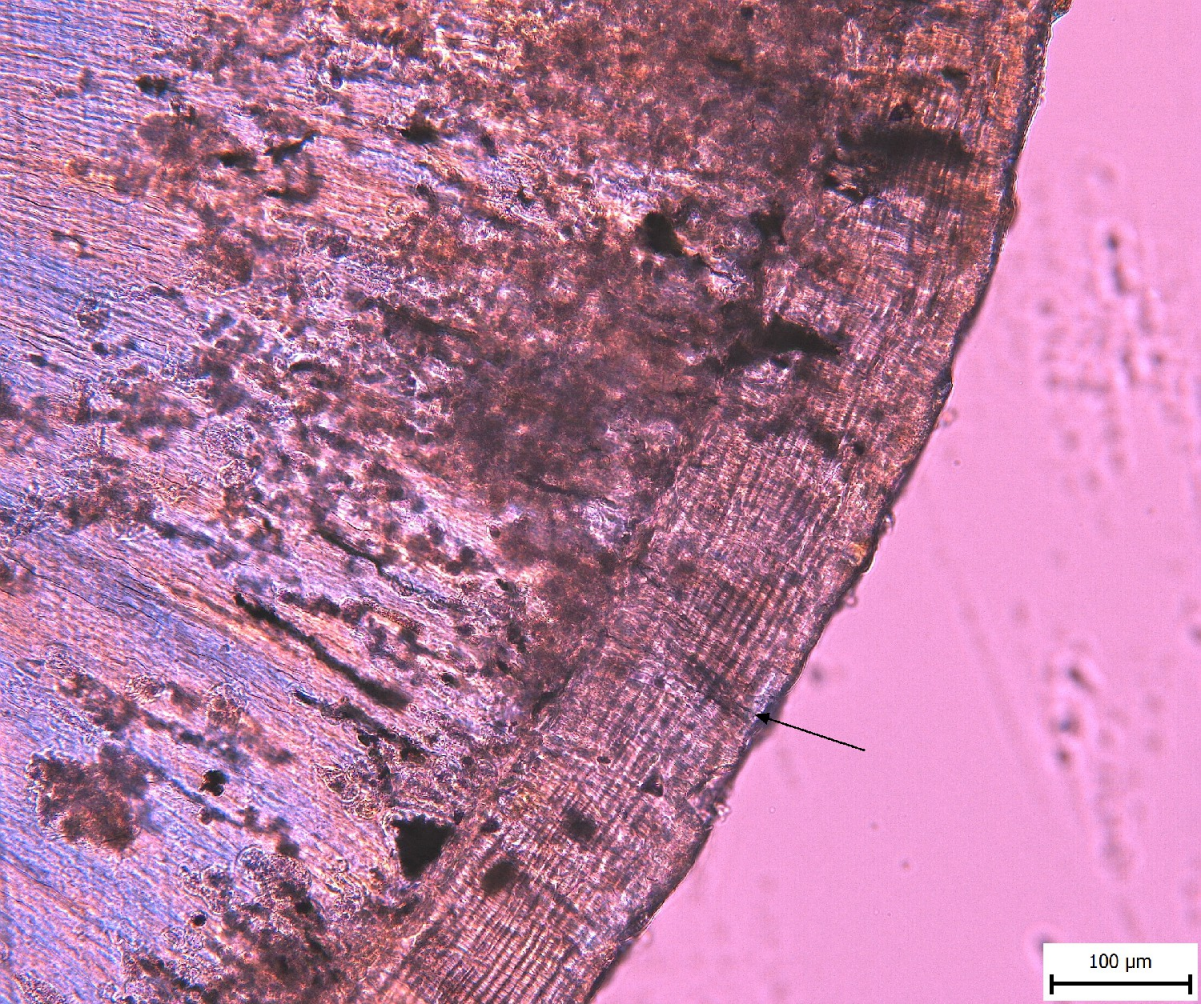
Thin section with readable cementum increments; the arrow indicates the translucent outermost increment.

The initial hypothesis was that two different seasons can be observed in the five examined mass graves. In the first examined group of mass graves (A and B, “plague” pits), the season-of-death estimation could be performed for 13 individuals. In 2 cases, the season was rather indeterminable, because of an unclear combination of bright and dark bands. In 11 individuals, the outermost line was bright, which corresponds with the spring/summer season (Table 1 and Fig 4). In the second groups of mass graves (C, D, E), the estimation could be performed for 15 individuals. In 3 of these, the season was indeterminable; in the remaining 12 individuals, the season-of-death estimation was autumn/winter, based on the dark outermost increment (Table 2).

**Table 1:** Season-of-death estimation based on cementochronology, mass graves A and B (“plague” mass graves/pits).

Skeleton number	Mass grave	Age (years)	Tooth type (FDI)	Season-of-death estimation
184	A	20–30	41	Spring/summer
212	A	23–30	41	Spring/summer
252	A	18–22	33	Indeterminable
257	A	15–17	43	Indeterminable
1426	A	14–16	32	Spring/summer
1428	A	20–25	33	Spring/summer
590	B	17–19	33	Spring/summer
591	B	20–40	43	Spring/summer
642	B	40–60	33	Spring/summer
812	B	20–30	43	Spring/summer
813	B	16–19	32	Spring/summer
814	B	20–25	43	Spring/summer
843	B	25–40	31	Spring/summer

**Table 2:** Season-of-death estimation based on cementochronology, mass graves C, D, E “famine” mass graves/pits).

Skeleton number	Mass grave	Age (years)	Tooth type (FDI)	Season estimation
585	C	25–50	13	Autumn/winter
625	C	18–22	11	Autumn/winter
627	C	40+	13	Autumn/winter
664	C	20–25	43	Indeterminable
810	C	30–50	33	Autumn/winter
826	C	20–25	33	Autumn/winter
828	C	17–19	33	Indeterminable
842	C	20–25	41	Autumn/winter
1250	C	30–50	32	Autumn/winter
1252	C	40–60	13	Autumn/winter
1253	C	18–20	42	Autumn/winter
800	D	25–50	33	Autumn/winter
820	D	40–60	33	Autumn/winter
1310	E	40–60	13	Indeterminable
1364	E	40–60	13	Autumn/winter

### Inter- and intra-observer error

Ten sections were randomly selected to assess inter-observer agreement in the identification of the outermost increment. One more experienced evaluator and one with no previous experience with cementochronology scored the same photos. Surprisingly, the agreement rate is high, in this case Cohen’s kappa equalling 0.9 (80%) (Table 3).

**Table 3:** Inter-observer agreement analysis.

Observation number	Observer 1	Observer 2	Agreement (Y/N)
1	bright	bright	Y
2	opaque	opaque?	Y
3	bright	bright?	Y
4	bright	bright	Y
5	opaque	opaque?	Y
6	bright	bright	Y
7	opaque	opaque?	Y
8	opaque	opaque	Y
9	bright	bright	Y
10	opaque	bright	Y

**Fig 5:**
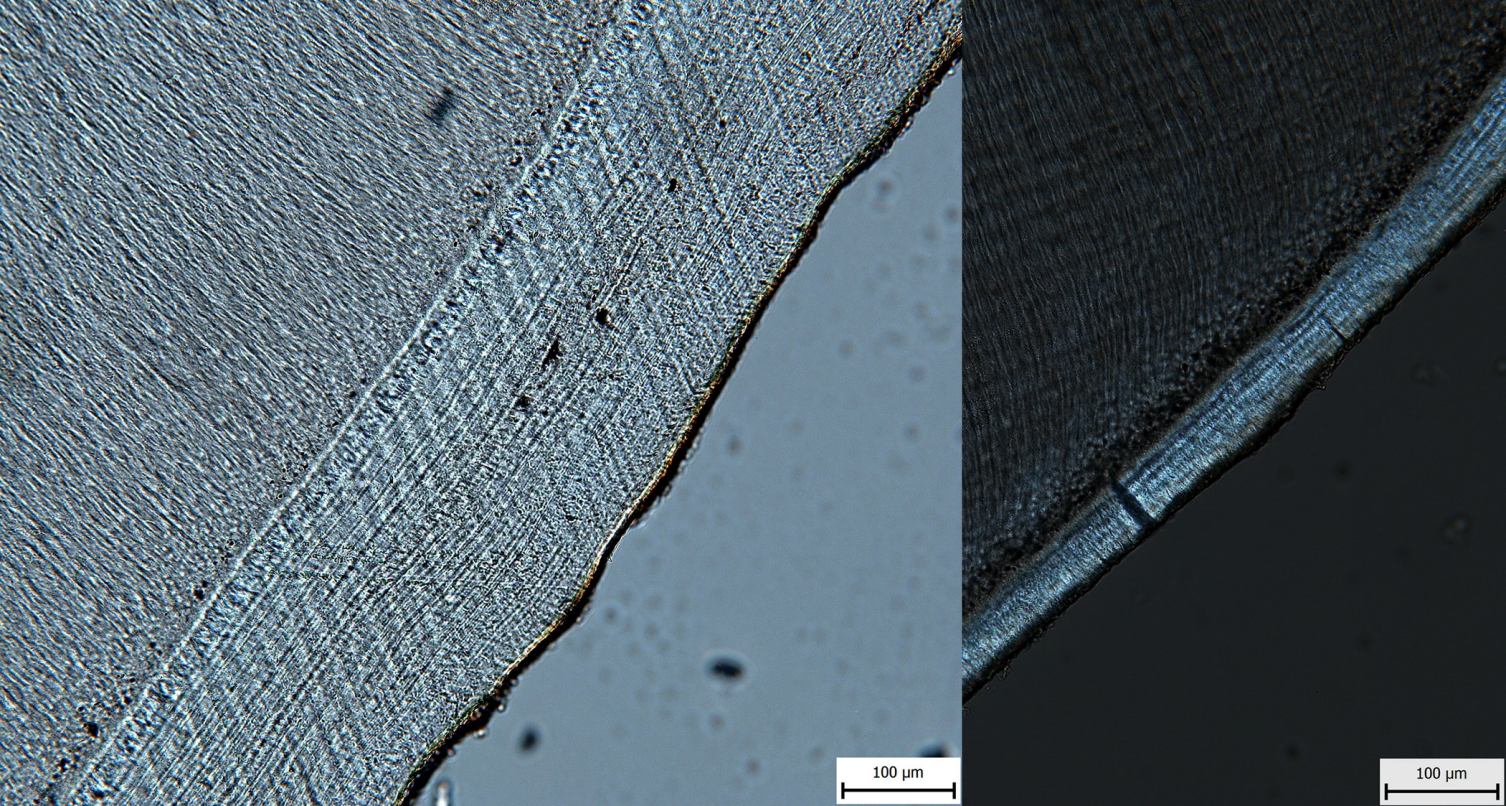
Comparison of the difficulty of assessing cementum increments in the context of age estimation and seasonality in individuals of different ages (left: 40–60 years, right: 17–19 years).

Ten sections were also randomly selected to assess intra-observer agreement in the identification of the outermost increment. In this case, the estimation is different for the three cases, and the agreement is therefore moderate (0.4). As in [45], the two originally dark lines have now been identified as light (Table 4). We also include a comparison of the level of difficulty in detecting the last increment according to the age of the individual (Fig 5).

**Table 4:** Intra-observer agreement analysis.

Observation number	Observation 1	Observer 2	Agreement (Y/N)
1	bright	bright	Y
2	opaque	opaque	Y
3	bright	bright	Y
4	bright	opaque	N
5	bright	bright	Y
6	bright	bright	Y
7	opaque	bright	N
8	opaque	bright	N
9	opaque	opaque	Y
10	opaque	opaque	Y

## Discussion

### Reconstructing seasonality using cementochronology: Technical notes

In our study, the season-of-death in 42 individuals from two stratigraphically distinct levels of mass graves was assessed using cementochronology. Our aim was to better understand the origin of the graves with special regard to the historical documents mentioning catastrophic events and related archaeological findings. We faced challenges in the evaluation that make the method a useful tool, but progress is needed in the protocol for evaluation or sampling. Although many studies indicate the potential of cementochronology for season-of-death estimation in anthropology (e. g., [46–49]), very few papers actually provide any results (e. g., [11,13,50]). The results of Wedel [12], with a 99% success rate for the method (distinguishing between two seasons: spring/summer and autumn/winter) sound very promising. Even so, since this study, there have been no other more numerous results that critically discuss evaluation procedures. Yet, there is a plethora of topics in bioarchaeology related to seasonality, and its assessment could enrich other indicators besides, for example, oxygen isotope analysis.

In archaeozoology, cementochronology is widely used for estimating the age and season of death; in bioarchaeology, the method of cementochronology generally meets with less involvement. Typically, the method serves as a useful tool for age-at-death estimation, although some critical studies have pointed out some limitations [7,51–54]. However, tooth cementum can be an advantageous indicator, especially for lifelong continuous tissue growth or generally good preservation of teeth in the archaeological record. Incremental lines in acellular cementum are considered to correlate with seasonal growth in most species [44]. Histological differences in the orientation of collagen fibres and their degree of mineralization are thought to explain the alternating light and dark increments in dental cementum [44,55]. Seasonal changes in diet, nutritional intake, hormonal cycles, sunlight or mastication are considered to be related factors [56]. What remains unclear is why, contrary to wild mammals, most modern humans and domestic mammals lacking a seasonal diet [45,57,58] still have regularly deposited cementum increments. The unclear etiology of the increments is often cited as one of the reasons why the method is not more widely used.

The application of cementochronology to estimate seasonality on human teeth is associated with several problematic factors, which we faced in this study. The terminology describing the outermost increment differs among researchers–the terms translucent and opaque are used (but with respect to the type of microscope in which a light increment may appear dark and vice versa), but also growth zone/annulus and slow/rapid growth [55]. In our study, the terms bright and dark increments are used. Human teeth are sometimes many times smaller than animal teeth, which makes the analysis more difficult. Observation can also be complicated by the large number of increments in individuals of advanced calendar age as well (Fig 5) [7]. This is a problem in anthropology [7], but also in archaeozoology [59]. Also, Wedel & Wescott [13] included only relatively young individuals in their study. There is also a relationship between the number of increments and tissue readability [7,60], with higher numbers of increments increasing the difference between chronological and estimated age. Regarding the archaeological material, the application of the method is moreover limited by diagenetic changes, possibly leading to a high percentage of discarded samples [61]. Another issue is related to the difference in detecting the two types of increments (translucent and opaque)—even in animals; the opaque line is more difficult to detect, because it is very thin [62]. In our case, it was also more difficult to identify the dark increment, which very often rather merged into the edge of the tooth, or remnants of fibres on it. To date, there are only a very small number of studies, few of which discuss inter- and intra-observer error, too. If this method is to be adopted for routine practice, it is necessary to expand the results. Compared to Wedel’s [12] study, Ralston [58] showed 61.5 to 71.2% correct season identification, and also 28.8% inter-observer error. Similarly, Thomas & Corby [63] demonstrated 58.3 to 73.9% correct season identification. Moreover, both studies faced the problem of identifying the true outermost increment, due to optical edge effects and inconsistencies in the outermost increments’ appearance. We too faced such issues, and agree with Thomas & Corby [63] that a modified protocol enhancing the outermost increment is strongly needed.

Good tissue visibility is critical, but may already be affected by age. This can be partly balanced by the preparation protocol, which should ensure the best possible result–with no cutting and grinding marks, and especially no glue marks on the tooth margin. Technical defects can be considered as reason for excluding samples [62]. However, it may still be the case that remnants of other tissues persisting on the tooth margin and partially cover the cementum. Finally, although some studies have shown no influence of periodontal disease on the results of age-at-death estimation [64,65], a no longer functional tooth can have disrupted apposition of the tissue, which can affect the results. Some pathological conditions (e. g. a fractured tooth, coronal destruction, caries) do not affect the results of the age estimation using cementochronology, but this does not apply to damaged desmodontium and periodontium, where they may render the tooth unusable for analysis or cause greater variation in age estimation [66]. Problems related to the preservation of tissues are unavoidable in an archaeological context; in bioarchaeology and forensic anthropology, however, the potential of season-of-death estimation is valuable and deserves further research. This may include a validation study in documented samples, larger samples balancing a high number of excluded teeth with diagenetic changes, or advanced technical steps such as measuring the outermost increment using measurement tools or considering the intra- and inter-observed error. Also, developing protocols for recording and presenting increment data would prevent researchers from subjectivity and counting false lines (caused by using different microscopy).

### Seasonality of famine and plague epidemics

The historical and archaeological evidence from Kutná Hora-Sedlec documents that some part of the population was impacted by serious catastrophes–episodes of famine (1318) and plague (1348–1350). Both events have specific seasonal dynamics, but their study in archaeology encompasses several limitations. Kutná Hora-Sedlec site is exceptional in terms of the number of individual and mass graves excavated during various excavation campaigns between 2010 and 2018, as part of restoration work on its famous ossuary chapel [15,16]. Individual graves (single inhumations) dated by radiocarbon dating belong to the 13-14^th^ and 14-16^th^ centuries, while the mass graves are dated to the 14^th^ century [68]. This period was a time of dramatic climate change for Bohemia (and Europe in general). While the 11^th^-13^th^ centuries were relatively warm and stable, around 1300 temperatures fell, and crop failures and famines occurred in the temperate zone. This gave rise to various negative phenomena, such as the mass migrations of people to cities and the emergence of diseases. The causes of the considerable die-off during the 14^th^ century at Kutná Hora-Sedlec are written in historical documents, which mention the victims of the famine of 1318. The so-called Great Famine is the best-documented famine, impacting northern and western Europe between 1315 and 1322 [67]. As for seasonality, famine episodes mostly occurred during the winter or early spring months [28–30]. Since famines were common among certain social groups in the Middle Ages, the famine must have been intense for it to appear in the chronicles, which are an invaluable source of information today [68]. Otherwise, it is true that hunger or the diet of the poor appeared rather rarely in medieval written sources [26]. Indeed, it is also very difficult to document famines on the basis of archaeological and osteological evidence alone [69]. Basically, two types of famine can be distinguished–acute and chronic. However, at the level of the skeletal remains, it is difficult to decide if an acute or chronic famine, or malnutrition, can be identified. In terms of osteological evidence, some signs associated with malnutrition (e. g. *cribra orbitalia*, Harris lines) [70] can be observed. These indicators are however non-specific, so they cannot be directly associated with famine (or e. g. infectious disease or other stress). The situation is also relativized by the osteological paradox, which draws attention to the important fact that an individual who dies of an acute disease, given that it is on the skeleton for a short duration, does not develop stress manifestations, and might be considered as healthy [71].

As regards the plague epidemic (1348–1350), we are faced with a lack of historical sources [21]. The main reason is that the plague epidemic was not as severe in Czechia as it was in, for example, in Italy or France. According to František Pražský, the plague reached Austria in 1349 and also began in Bohemia. Also, the first attack of the plague was pushed out by “fresh and cold air” [20]. This would be in line with information about known plague seasonal dynamics. On the basis of being related to latitude, temperature and precipitation, the epidemic growth of plague outbreaks was positive between 11.7°C and 21.5°C, and second pandemic outbreaks peaked in the summer [23,32]. The problem is that there is no completely accurate meteorological data, so we have to work with model data. Mild winters or short-term fluctuations are difficult to reflect. While there are possibilities to distinguish between famine and plague graves on the basis of osteological evidence alone (without archaeogenetic analyses), options are limited. The situation is further complicated by the fact that there are situations in which a population faced the famine and plague epidemics at the same time [21,72]. Catastrophes such as wars or disease may lead to a change in funerary rite, and also result in specific features in the skeletal population [73]. At the level of a group of individuals (or population), a specific (attritional) mortality profile that differs from the normal one can support the identification of a catastrophic event [73–75]. Compared to attritional mortality, where the highest risk of death is for infants and older adults, catastrophic mortality should mimic the age structure of the living population, having a high risk of death in all age categories [75–77]. In this case too, great attention should be given to the choice of age estimation methods, e.g. an excess number of middle-aged adults may affect the mortality curve and its interpretation [75]. Interestingly, in the study of Lépinau et al. [17], the examined mass graves (numbers 765, 853 and 1578, in our study: C, D, E) were assigned more to plague mortality, on the basis of the age structure and pattern of skeletal lesions. Trying to find a specific age pattern for catastrophic events (mainly plague epidemics) is the subject of research [75–78], the mortality pattern of the 14^th^ century Black Death was not very different from normal medieval mortality, and plague preferentially killed older adults and individuals with poor health. Together with our findings it is clear that additional research including archaeogenetic analyses [79] is needed to clear up the nature of mass graves in Kutná Hora-Sedlec. In conclusion, the results of season-of-death estimation using cementochronology identified two different seasons in the investigated mass graves. This makes the method a useful tool, for example, in cases where a question related to seasonality is being addressed on archaeological material.

## Conclusions

Cementochronology has so far rarely been applied in bioarchaeology to estimate the season-of-death. The reasons may vary, but in the context of explanatory value, the method can potentially address issues related to seasonality. In our study, we managed to identify two different burial periods which can hypothetically be associated with catastrophic events known from historical and archaeological records. It is impossible to say whether a specific catastrophic event was directly involved based on season-of-death estimation alone. Still, our findings contribute to understanding the differences between the two groups of graves, and to discussing the results with a knowledge of the seasonality of famine figand plague. Thus, cementochronology could also be applied in bioarchaeology, but with inevitable (diagenetic changes) and avoidable limitations (selection of younger and middle-aged adults, emphasis on standardization of procedures, maximum removal of observer subjectivity).

## Supporting information

S1 Table Readability index values for the assessed thin sections. (DOCX)

S2 Table Season-of-death estimations for all mass graves. (DOCX)
