# Supplementary material for: Using cementochronology to assess the seasonality of catastrophic events in medieval mass graves (Kutná Hora-Sedlec, Czechia, 14th century): Preliminary results
Source: PLoS One. 2023 Dec 13;18(12):e0295757. doi: 10.1371/journal.pone.0295757 (PMC10718420; doi:10.1371/journal.pone.0295757)
Supplement: S1 Table — (DOCX) [file pone.0295757.s001.docx]

| **Skeleton number** | **Mass grave number** | **Mass grave code** | **Tooth type (FDI)** | **R_index_1** | **R_index_2** | **R_index_3** | **R_index_4** | **R_index_5** |
| --- | --- | --- | --- | --- | --- | --- | --- | --- |
| 98 | 516 | A | 13 | 0 | 0 | 0 | 0 | 0 |
| 184 | 516 | A | 41 | 3 | 3 | 2 | 3 | 2 |
| 212 | 516 | A | 41 | 3 | 3 | 2 | 2 | 2 |
| 252 | 516 | A | 33 | 3 | 3 | 2 | 2 | 0 |
| 257 | 516 | A | 43 | 1 | 0 | 0 | 0 | 0 |
| 934 | 516 | A | 31 | 0 | 0 | 0 | 0 | 0 |
| 1426 | 516 | A | 32 | 3 | 2 | 0 | 0 | 2 |
| 1428 | 516 | A | 33 | 2 | 2 | 2 | 1 | 0 |
| 533 | 764 | B | 22 | 0 | 0 | 0 | 0 | 0 |
| 557 | 764 | B | 13 | 0 | 0 | 0 | 0 | 0 |
| 565 | 764 | B | 12 | 0 | 0 | 0 | 0 | 0 |
| 567 | 764 | B | 43 | 0 | 0 | 0 | 0 | 0 |
| 589 | 764 | B | 43 | 0 | 0 | 0 | 0 | 0 |
| 590 | 764 | B | 33 | 2 | 2 | 0 | 0 | 0 |
| 591 | 764 | B | 43 | 3 | 3 | 3 | 3 | 3 |
| 611 | 764 | B | 33 | 0 | 0 | 0 | 0 | 0 |
| 642 | 764 | B | 33 | 0 | 2 | 2 | 0 | 0 |
| 738 | 764 | B | 43 | 0 | 0 | 0 | 0 | 0 |
| 812 | 764 | B | 43 | 0 | 0 | 0 | 0 | 1 |
| 813 | 764 | B | 32 | 1 | 0 | 0 | 0 | 0 |
| 814 | 764 | B | 43 | 2 | 2 | 2 | 0 | 0 |
| 843 | 764 | B | 31 | 0 | 3 | 3 | 0 | 2 |
| 585 | 765 | C | 13 | 2 | 2 | 0 | 0 | 0 |
| 586 | 765 | C | 43 | 0 | 0 | 0 | 0 | 0 |
| 625 | 765 | C | 11 | 1 | 2 | 2 | 0 | 0 |
| 627 | 765 | C | 13 | 2 | 2 | 0 | 2 | 2 |
| 664 | 765 | C | 43 | 0 | 0 | 0 | 0 | 1 |
| 777 | 765 | C | 33 | 0 | 0 | 0 | 0 | 0 |
| 810 | 765 | C | 33 | 2 | 2 | 0 | 0 | 0 |
| 825 | 765 | C | 23 | 0 | 0 | 0 | 0 | 0 |
| 826 | 765 | C | 33 | 0 | 0 | 0 | 2 | 2 |
| 828 | 765 | C | 33 | 2 | 2 | 2 | 0 | 0 |
| 842 | 765 | C | 41 | 2 | 2 | 2 | 0 | 0 |
| 1249 | 765 | C | 43 | 0 | 0 | 0 | 0 | 0 |
| 1250 | 765 | C | 32 | 2 | 0 | 2 | 0 | 0 |
| 1252 | 765 | C | 13 | 2 | 2 | 2 | 0 | 0 |
| 1253 | 765 | C | 42 | 2 | 2 | 2 | 0 | 0 |
| 1256 | 765 | C | 43 | 0 | 0 | 0 | 0 | 0 |
| 800 | 853 | D | 33 | 0 | 0 | 0 | 0 | 1 |
| 820 | 853 | D | 33 | 2 | 2 | 1 | 2 | 2 |
| 1310 | 1578 | E | 13 | 2 | 0 | 0 | 0 | 0 |
| 1364 | 1578 | E | 13 | 0 | 0 | 2 | 0 | 0 |
|  |  |  |  |  |  |  |  |  |
| *FDI = Fédération Dentaire Internationale, R_index = readability index (scale 0-4) | | | | | | | | |
