# Supplementary material for: Using cementochronology to assess the seasonality of catastrophic events in medieval mass graves (Kutná Hora-Sedlec, Czechia, 14th century): Preliminary results
Source: PLoS One. 2023 Dec 13;18(12):e0295757. doi: 10.1371/journal.pone.0295757 (PMC10718420; doi:10.1371/journal.pone.0295757)
Supplement: S2 Table — (DOCX) [file pone.0295757.s002.docx]

| **skeleton number** | **mass grave number** | **season_est_1** | **season_est_2** | **season_est_3** | **season_est_4** | **season_est_5** | **season_est** |
| --- | --- | --- | --- | --- | --- | --- | --- |
| 98 | 516 | N/A | N/A | N/A | N/A | N/A | N/A |
| 184 | 516 | bright | bright | bright | bright | bright | spring/summer |
| 212 | 516 | bright | bright | bright | dark | bright | spring/summer |
| 252 | 516 | dark | dark | bright | bright | N/A | indeterminable |
| 257 | 516 | bright? | N/A | N/A | N/A | N/A | indeterminable |
| 934 | 516 | N/A | N/A | N/A | N/A | N/A | N/A |
| 1426 | 516 | bright | bright | bright | N/A | N/A | spring/summer |
| 1428 | 516 | bright | bright | bright | bright | bright | spring/summer |
| 533 | 764 | N/A | N/A | N/A | N/A | N/A | N/A |
| 557 | 764 | N/A | N/A | N/A | N/A | N/A | N/A |
| 565 | 764 | N/A | N/A | N/A | N/A | N/A | N/A |
| 567 | 764 | N/A | N/A | N/A | N/A | N/A | N/A |
| 589 | 764 | N/A | N/A | N/A | N/A | N/A | N/A |
| 590 | 764 | bright | bright | N/A | N/A | N/A | spring/summer |
| 591 | 764 | bright | bright | bright | bright | bright | spring/summer |
| 611 | 764 | N/A | N/A | N/A | N/A | N/A | N/A |
| 642 | 764 | N/A | bright | bright | N/A | N/A | spring/summer |
| 738 | 764 | N/A | N/A | N/A | N/A | N/A | N/A |
| 812 | 764 | N/A | N/A | N/A | bright | bright | spring/summer |
| 813 | 764 | bright | N/A | N/A | N/A | N/A | spring/summer |
| 814 | 764 | bright | bright | bright | N/A | N/A | spring/summer |
| 843 | 764 | N/A | bright | bright | N/A | bright | spring/summer |
| 585 | 765 | dark | dark | dark | N/A | N/A | autumn/winter |
| 586 | 765 | N/A | N/A | N/A | N/A | N/A | N/A |
| 625 | 765 | dark | dark | dark | N/A | N/A | autumn/winter |
| 627 | 765 | dark | dark | N/A | dark | dark | autumn/winter |
| 664 | 765 | dark | N/A | N/A | N/A | dark | indeterminable |
| 777 | 765 | N/A | N/A | N/A | N/A | N/A | N/A |
| 810 | 765 | dark | dark | N/A | N/A | N/A | autumn/winter |
| 825 | 765 | N/A | N/A | N/A | N/A | N/A | N/A |
| 826 | 765 | N/A | N/A | N/A | dark | dark | autumn/winter |
| 828 | 765 | bright | dark | dark | N/A | N/A | indeterminable |
| 842 | 765 | dark | dark | dark | N/A | N/A | autumn/winter |
| 1249 | 765 | N/A | N/A | N/A | N/A | N/A | N/A |
| 1250 | 765 | dark | N/A | dark | N/A | N/A | autumn/winter |
| 1252 | 765 | dark | dark | dark | N/A | N/A | autumn/winter |
| 1253 | 765 | dark | dark | dark | N/A | N/A | autumn/winter |
| 1256 | 765 | N/A | N/A | N/A | N/A | N/A | N/A |
| 800 | 853 | N/A | N/A | N/A | N/A | N/A | autumn/winter |
| 820 | 853 | dark | N/A | dark | N/A | N/A | autumn/winter |
| 1310 | 1578 | dark? | N/A | N/A | N/A | N/A | indeterminable |
| 1364 | 1578 | dark | dark | dark | N/A | N/A | autumn/winter |
